# Knowledge and Attitudes Toward Sexual and Reproductive Health Among Adolescents Studying in Government Schools in Tansen Municipality, Palpa, Nepal: A School‐Based Cross‐Sectional Study

**DOI:** 10.1002/puh2.70305

**Published:** 2026-06-23

**Authors:** Sangita Bhattarai, Shrijana Poudel, Krishna Prasad Sapkota, Rais Pokharel, Bikash Adhikari, Bhuwan Thakurathi, Rajan Bikram Rayamajhi, Nilam Adhikari

**Affiliations:** ^1^ Tribhuvan University, Institute of Medicine, Pokhara Nursing Campus Pokhara Nepal; ^2^ Department of Sociology and Gerontology Miami University Oxford Ohio USA; ^3^ World Health Organization Country Office for Nepal Kathmandu Nepal; ^4^ Purbanchal University Teaching Hospital Morang Nepal; ^5^ Ministry of Health and Population, Government of Nepal Kathmandu Nepal; ^6^ Freelance Researcher Kathmandu Nepal; ^7^ School of Public Health and Community Medicine BP Koirala Institute of Health Sciences Dharan Nepal

**Keywords:** reproductive health, sexual health, South Asia, teenagers

## Abstract

**Background:**

Adolescents’ sexual and reproductive health (SRH) knowledge and attitudes are critical determinants of future health behaviors and service utilization. In Nepal, despite the inclusion of comprehensive sexuality education (CSE) within the national curriculum, evidence suggests persistent gaps in SRH knowledge and unfavorable attitudes among school‐going adolescents, particularly in public school settings. This study aimed to assess the level of SRH knowledge and attitudes and factors associated with favorable SRH attitudes among adolescents.

**Methods:**

A school‐based cross‐sectional study was conducted among adolescents aged 10–19 years enrolled in Grades 8–10 in three purposively selected government schools in Tansen Municipality, Palpa District. Data were collected using a structured, self‐administered questionnaire covering SRH knowledge domains (puberty, abortion, sexually transmitted infections, and family planning) and SRH attitudes. SRH knowledge was categorized as poor, moderate, and good for respective domains. Likewise, attitude was measured using 10 Likert‐scale statements and categorized as favorable or unfavorable. Descriptive statistics summarized knowledge and attitudes, whereas binary and multivariable logistic regression analyses were used to identify factors associated with favorable SRH attitudes. Results are presented in adjusted odds ratios (AOR) and 95% confidence intervals (CI).

**Results:**

Among the 228 participants, moderate knowledge was most common across SRH domains, whereas good knowledge remained limited. Nearly two‐thirds (62.3%) of adolescents demonstrated unfavorable SRH attitudes. Adolescents with moderate (AOR = 4.41, 95% CI: 1.09–17.83) and good knowledge of abortion (AOR: 4.93; 95% CI: 1.35–17.95; *p* = 0.016) were significantly more likely to have favorable SRH attitudes compared with those with poor knowledge.

**Conclusion:**

Lower percentage of adolescents with good SRH knowledge and higher percentage with unfavorable attitudes highlight gaps in SRH education in government schools. CSE, including abortion‐related education, could be prioritized in government schools to uplift the SRH knowledge and attitudes.

## Graphical abstract

A school‐based cross‐sectional study in western Nepal found substantial gaps in adolescents’ sexual and reproductive health knowledge and predominantly unfavorable attitudes. Abortion‐related knowledge showed a significant independent association with favorable SRH attitudes, highlighting domain‐specific influences on adolescents’ perceptions.

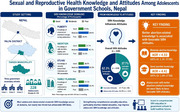

## 1 Introduction

Adolescents are defined as those aged 10–19 years, representing a prime segment of the global population that has faced considerable challenges related to sexual and reproductive health (SRH) [1]. Despite increased global attention following the 1994 International Conference on Population and Development (ICPD), which promoted a rights‐based approach to adolescent SRH, many adolescents continue to face limited access to accurate information and essential services, particularly in low‐ and middle‐income countries (LMICs) [2, 3]. Each year, an estimated 12.8 million births occur among girls aged 15–19, alongside approximately 3 million unsafe abortions [4, 5]. Furthermore, an estimated 2.1 million adolescents are living with HIV, with half of infection occurring within this age group [6, 7]. In LMICs, approximately 23 million adolescents have an unmet need for family planning [8], and comprehensive knowledge of HIV/AIDS remains low; for instance, only 20% of girls and 36% of boys aged 15–19 in India are well‐informed on the subject [9].

In Nepal, adolescents represent 24% of the total population [10]. According to the Nepal Demographic and Health Survey (2016), 17% of adolescent girls have already begun childbearing, and 4% of married girls aged 15–19 already use modern contraceptive methods [11]. Early sexual activity increases the risk of unintended pregnancies and sexually transmitted infections (STIs). Community‐based studies in Nepal highlighted that nearly half of adolescents possess only moderate knowledge of SRH topics [12].

Despite being such a prime and critical population group, adolescents in Nepal continue to face major barriers in accessing accurate SRH information and services. Cultural taboos, limited coverage in educational curricula, discomfort among educators, deep‐rooted community level stigma, travel‐related access issues, barriers within healthcare, concerns about privacy and confidentiality, and unprofessional attitudes from staff all contribute to further hinder their ability to make informed health decisions [13]. Addressing these issues requires further community‐based research to guide evidence‐based interventions and strengthen Nepal's alignment with national and global adolescent SRH priorities.

Previous studies in Nepal have assessed adolescents’ general SRH knowledge and awareness [12, 14, 15]. However, limited evidence has examined how different SRH knowledge domains relate to adolescents’ attitudes, particularly among government‐school adolescents in semi‐urban areas of western Nepal [16, 17]. This study addresses this gap by assessing domain‐specific SRH knowledge and its association with SRH attitudes among adolescents in Tansen Municipality, Nepal.

This study assessed the knowledge and attitude levels on SRH among school‐going adolescents in Nepal. It evaluated the adolescents’ understanding across key SRH domain, namely, pubertal changes, abortion, STIs, and family planning. Additionally, this study assessed adolescents’ attitudes toward SRH and examined associations between knowledge levels and sociodemographic factors to identify predictors of favorable SRH attitudes.

## 2 Methods

This study has been reported following STORBE guidelines for cross‐sectional studies (uploaded it as a Supporting Information).

### 2.1 Study Design and Setting

We conducted a school‐based cross‐sectional study to assess knowledge and attitudes toward SRH among school‐going adolescents in Tansen Municipality located in Palpa District of Lumbini Province, western Nepal. Tansen is the administrative headquarters of Palpa district and is nestled in the mid‐hill region of the country [18]. According to the National Population and Housing Census, 2021, Tansen has a total population of 50,792, of which 18.1% fall within the adolescent age group (10–19 years) [19]. The municipality comprises both urban and semi‐urban areas and hosts multiple government schools where adolescents from diverse ethnic and socioeconomic backgrounds are enrolled.

### 2.2 Sample Size and Sampling Technique

Three government secondary schools were purposively selected from the 18 government schools in Tansen Municipality, Palpa. The schools were selected to capture adolescents from different catchment areas within the municipality and based on administrative feasibility, student enrollment size, and permission for data collection during the study period. Government schools were chosen because they enroll adolescents from diverse socioeconomic and ethnic backgrounds and follow the national curriculum.

After selecting the schools, a census method was used, where all eligible students from Grades 8 to 10 who were present during the data collection period were invited to take part in the study. Eligible participants were adolescents aged 10–19 years enrolled in the selected schools and grades. Students who were absent during data collection or who refused to participate were excluded.

Sample size was calculated using the formula for estimating single sample population formula for finite population (*n* = *Z* ^2^ *pq*/*e* ^2^), where *Z* = 1.96 (95% confidence level), *p* = 0.628 (estimated prevalence of knowledge 62.8% [20]), and *e* = 0.05 (margin of error). The initial estimated sample size was 360. As the total eligible student population in the selected schools was 574, a finite population correction was applied, resulting in an adjusted sample size of 222. After adding 10% to account for possible nonresponse, the final estimated sample size was 244. On the day of data collection, 16 eligible students were absent, leading to a final sample size of 228 participants.

### 2.3 Data Collection Instrument

A structured, self‐administered questionnaire was developed on the basis of an extensive literature review and International Technical Guidance on Sexuality Education published by UNICEF, UN Women, and the World Health Organization (WHO) [21]. The instrument was designed to collect information on sociodemographic background, knowledge, and attitudes regarding SRH among adolescents. The questionnaire was divided into three sections:

- **Part I** focused on sociodemographic information and included six items related to age, sex, grade, ethnicity, parental education, and sources of SRH information.
- **Part II** assessed knowledge on SRH and comprised 24 items. These covered four thematic areas: pubertal changes (five items), abortion (eight items), STIs (seven items), and family planning (four items).
- **Part III** measured attitudes toward SRH through 10 statements, each rated on a five‐point Likert scale ranging from “strongly agree” to “strongly disagree.”

Full details of the questionnaire are available in the Supporting Information.

The questionnaire was initially developed in English and then translated into Nepali to ensure clarity and cultural appropriateness. A back‐translation into English was performed by a separate translator to verify consistency and accuracy.

The internal consistency reliability of the study instruments was assessed using Cronbach's alpha coefficient. The overall SRH knowledge scale demonstrated good reliability (*α* = 0.87). Domain‐specific reliability estimates were acceptable for abortion knowledge (*α* = 0.74) and STI knowledge (*α* = 0.68), whereas pubertal changes (*α* = 0.54) and family planning knowledge (*α* = 0.59) showed lower internal consistency. The SRH attitude scale demonstrated low internal consistency (*α* = 0.48), indicating that the attitude items may not reliably measure a single underlying construct.

To ensure content validity, the tool underwent expert review and iterative refinement based on feedback from the research guide and peers. A pretest was conducted with 23 students from Grade 10 at similar setting of Amarsingh Secondary School to assess clarity, feasibility, and time requirements for administration.

Data collection was conducted between 13 June 2019 and 25 June 2019. All participants were adolescents aged 10–19 years enrolled in Grades 8–10 of the selected government schools. As the study involved minors, written informed consent was obtained from parents or legal guardians. On the first day, the investigator distributed the Nepali‐language consent forms to students, who then took them home for signature by their parents/guardians. Only students who returned signed consent forms were enrolled in the study. In addition, verbal assent was obtained from the students before administering the questionnaire.

A structured, self‐administered questionnaire was given to eligible students during regular school hours in their classrooms. Class teachers supported the process only by helping coordinate the timetable and organize students. They were not involved in distributing the questionnaires, supervising students while they completed them, or viewing any of the responses.

Data were collected directly by the research team to maintain confidentiality and minimize the risk of social desirability bias.

Completion of the questionnaire took 25–30 min, under the direct supervision of the principal investigator. Prior to participation, students were informed about the voluntary nature of the study and assured of their right to withdraw at any stage.

### 2.4 Ethical Consideration

This study was conducted in accordance with the principles of the Declaration of Helsinki and Nepal's national ethical guidelines from Nepal Health Research Council. Ethical approval was obtained from the Pokhara Nursing Campus, Institute of Medicine, Tribhuvan University (Reference no. 807/075/076), as part of the requirements for the completion of a Bachelor of Nursing Science thesis. Additional administrative approvals were obtained from the Tansen Municipality, its respective Ward Offices, and the selected government schools.

Informed written consent was obtained from the parents after thoroughly explaining the study objectives, procedures, potential risks, and benefits. In addition, verbal assent was obtained from all adolescent participants before administration of the questionnaire. Participant confidentiality and anonymity were strictly maintained by assigning unique codes and removing any identifying personal information. Data were securely stored and used solely for research purposes. Respondents were assured of their right to withdraw at any point without any consequences or loss of benefit.

### 2.5 Measures

#### 2.5.1 Knowledge of SRH

Knowledge of SRH was assessed using a structured questionnaire comprising 24 items across four domains: pubertal changes, abortion, STIs, and family planning. Some items were single response options, and some were multiple responses. Each item was scored dichotomously (1 = correct, 0 = incorrect), and for multiple‐response items, each correct response option was given one point without penalizing incorrect responses. The pubertal changes comprise 5 items and have total 17 points in total, abortion (8 items, 24 points), STIs (7 items, 30 points), and family planning (4 items, 16 points).

Each domain‐wise total knowledge scores were calculated, then converted into percentages, and finally categorized using the thresholds of good knowledge (≥75% correct), moderate knowledge (50%–74%), and poor knowledge (<50%) [22]. This classification enabled a standardized comparison and identification of knowledge gaps among adolescents.

The number of items differed across SRH knowledge domains; therefore, raw domain scores were converted into percentage scores by dividing each participant's obtained score by the maximum possible score for that domain and multiplying by 100. This approach ensured standardization across domains and allowed meaningful comparison despite unequal score ranges.

#### 2.5.2 Attitudes on SRH

Attitude toward SRH was assessed using 10 statements rated on a five‐point Likert scale, ranging from “strongly disagree” (1) to “strongly agree” (5). The items captured adolescents’ perceptions toward contraceptive use, SRH education, STI prevention, menstruation, and the role of parents in information‐sharing. Among these, negatively worded statements—such as “Contraceptive use for a long period causes infertility,” “Providing emergency contraceptive pills would discourage consistent use of other contraceptives,” “Menstruation is a shameful and embarrassing situation for girls,” and “Adolescents do not need SRH information because they have no premarital intercourse”—were reverse‐coded so that a higher score consistently reflected a more favorable attitude. The total attitude score ranged from 10 to 50. On the basis of the modified Bloom's cut‐off point, ≥75% of the scores (score ≥38) were categorized as “favorable attitude” and scores <38 as “unfavorable attitude” for subsequent analysis [23]. This threshold was applied to maintain consistency with commonly used knowledge–attitude–practice (KAP) classification approaches and to reflect a higher level of positive SRH attitudes.

### 2.6 Conceptual Framework

This study was guided by a conceptual framework adapted from the KAP model, which posits that knowledge influences attitudes, which, in turn, shape behaviors. In this framework, adolescents’ SRH attitudes were considered the primary outcome variable.

SRH knowledge was conceptualized across four domains: pubertal changes, abortion, STIs, and family planning. These knowledge domains were hypothesized to directly influence adolescents’ attitudes toward SRH.

In addition, sociodemographic factors, including age, sex, grade, ethnicity, and parental education, were included as background variables that may independently or indirectly influence SRH attitudes.

### 2.7 Operational Definition

Adolescents were categorized following into two age groups: early adolescents (10–14 years) and late adolescents (15–19 years) [24]. Ethnicity was self‐reported and classified into Janajati (indigenous groups), Brahmin/Chhetri (dominant caste groups), and Dalit (historically marginalized communities) [25]. Parental education was grouped into four levels: illiterate (no formal schooling), primary education (up to Grade 5), secondary education (Grades 6–10), and SLC and above (School Leaving Certificate/Grade 10 or higher).

Sources of information on SRH were captured through multiple response options, including teachers, radio/television, parental education, peers, and siblings.

### 2.8 Quantitative Variables

Quantitative variables included age, SRH knowledge scores, and SRH attitude scores. Age was categorized into early adolescence (10–14 years) and late adolescence (15–19 years), as defined by the United Nations classification [24].

For each SRH knowledge domain (pubertal changes, abortion, STIs, and family planning), raw knowledge scores were calculated by assigning one point for each correct response and zero points for incorrect or “don't know” responses. Domain‐specific raw scores were converted into percentage scores by dividing the obtained score by the maximum possible score for that domain and multiplying by 100. Percentage scores were then categorized as good (≥75), moderate (50–74), or poor (<50) knowledge using the modified Bloom's cut‐off points.

Attitudes toward SRH were measured using 10 Likert‐scale statements scored from 1 (strongly disagree) to 5 (strongly agree). Negatively worded items were reverse‐coded so that higher scores consistently represented more favorable attitudes. Total attitude scores ranged from 10 to 50 and were converted into percentage scores. Using the modified Bloom's cut‐off point, scores ≥75% (≥38 out of 50) were classified as favorable attitude, whereas scores <75% were classified as unfavorable attitude. These categorical variables were used in descriptive analyses and logistic regression to identify factors associated with favorable SRH attitudes.

### 2.9 Statistical Analysis

The collected data were compiled, cleaned, and checked for completeness and consistency in Microsoft Excel 2016 and then exported to SAS software version 9.4 (SAS Institute Inc., Cary, NC, USA) for statistical analysis. Descriptive statistics, including frequencies and percentages, were used to summarize participants’ sociodemographic characteristics and key study variables. Associations between independent variables and SRH attitude (favorable vs. unfavorable) were examined using binary logistic regression. Variables with *p* < 0.20 in the bivariate analysis were entered into a multivariable logistic regression model to identify factors independently associated with favorable SRH attitudes. Crude odds ratios (COR) and adjusted odds ratios (AOR) with 95% confidence intervals (CIs) were reported, and statistical significance was set at *p* < 0.05. Due to high collinearity between age and school grade, grade was excluded from the final multivariable model to improve model stability. Afterwards, multicollinearity among independent variables was examined using the variance inflation factor (VIF), and no evidence of multicollinearity was observed (all VIF values <2.5).

Model fit was assessed using the Hosmer–Lemeshow goodness‐of‐fit test, which indicated adequate fit (*χ* ^2^(8) = 9.04, *p* = 0.34). Model discrimination was evaluated using the concordance statistic (*c*‐statistic), which showed acceptable predictive performance (*c* = 0.68). The explanatory power of the final model was assessed using Nagelkerke's pseudo *R* ^2^, which was 0.13, indicating a modest proportion of variance explained.

## 3 Results

### 3.1 Sociodemographic Characteristics of the Respondents

A total of 228 adolescent students participated in the study. The sociodemographic characteristics of the respondents are presented in Table 1. The mean age of the respondents was 14.17 years (SD ±1.35). The majority were early adolescents aged 10–14 years (64.0%), whereas 36.0% were late adolescents aged 15–19 years. Nearly half of the participants were enrolled in Grade 10 (45.61%), followed by Grades 8 (28.95%) and 9 (25.44%).

**TABLE 1:** Sociodemographic characteristics of the respondents (*n* = 228).

Characteristics	Number (*n*)	Percentage
**Age group (in years)**		
Early adolescent	146	64.00
Late adolescent	82	36.00
**Mean age/SD (14.17 ± 1.35)**		
**Grade**		
8	66	28.95
9	58	25.44
10	104	45.61
**Gender**		
Female	119	52.20
Male	109	47.80
**Ethnicity**		
Janajati	107	46.90
Brhman/Chhetri	72	31.60
Dalit	49	21.50
**Education of mother**		
Illiterate	40	17.50
Primary education	70	30.70
Secondary education	71	31.20
SLC and above	47	20.60
**Education of father**		
Illiterate	16	7.00
Primary education	59	25.90
Secondary education	90	39.50
SLC and above	63	27.60
**Source of information** ^a^		
Teachers	180	78.90
Radio/Television	123	53.90
Parent's education	84	36.80
Peers	64	28.10
Siblings	29	12.70

^a^ Multiple response question.

In terms of sex distribution, 52.20% of the respondents were female and 47.80% were male. Almost half of the adolescents belonged to Janajati groups (46.90%), followed by Brahmin/Chhetri (31.60%) and Dalit (21.50%).

With respect to parental education, 31.20% of mothers had completed secondary education, whereas 30.70% had primary education and 17.50% were illiterate. Among fathers, 39.50% had completed secondary education, 27.60% had attained SLC or higher education, and only 7.00% were illiterate.

Teachers were reported as the most common source of SRH information (78.90%), followed by radio or television (53.90%). Other sources included parents (36.80%), peers (28.10%), and siblings (12.70%).

### 3.2 Knowledge and Attitudes Toward SRH

**FIGURE 1:**
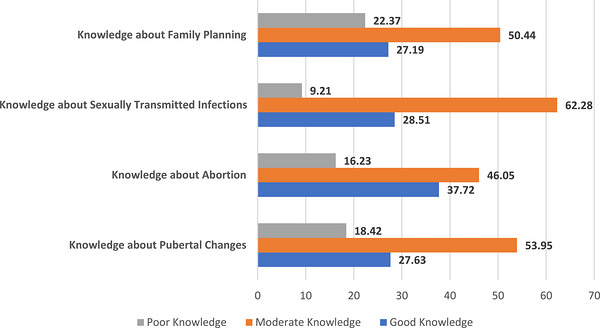
Distribution of sexual and reproductive health knowledge levels among respondents (*n* = 228).

The distribution of overall SRH knowledge levels among respondents is presented in Figure 1. Table 2 presents adolescents’ levels of knowledge across four domains of SRH and their overall attitudes toward SRH. Knowledge of pubertal changes was predominantly moderate, with 53.95% of respondents demonstrating moderate knowledge, whereas 27.63% had good knowledge and 18.42% exhibited poor knowledge.

**TABLE 2:** Knowledge level of SRHR among respondents (*n* = 228).

Characteristics	Number (*n*)	Percent
**Knowledge about pubertal changes**		
Good knowledge	63	27.63
Moderate knowledge	123	53.95
Poor knowledge	42	18.42
**Knowledge about abortion**		
Good knowledge	86	37.72
Moderate knowledge	105	46.05
Poor knowledge	37	16.23
**Knowledge about sexually transmitted infections**		
Good knowledge	65	28.51
Moderate knowledge	142	62.28
Poor knowledge	21	9.21
**Knowledge about family planning**		
Good knowledge	62	27.19
Moderate knowledge	115	50.44
Poor knowledge	51	22.37
**Attitude about sexual and reproductive health**		
Unfavorable attitude	142	62.28
Favorable attitude	86	37.72

Regarding abortion, nearly half of the respondents (46.05%) had moderate knowledge, and more than one‐third (37.72%) demonstrated good knowledge; however, 16.23% of adolescents had poor knowledge in this domain. Knowledge related to STIs was comparatively higher, with 62.28% of respondents showing moderate knowledge and 28.51% demonstrating good knowledge, whereas only 9.21% had poor knowledge.

In contrast, knowledge of family planning was relatively limited. Half of the respondents (50.44%) had moderate knowledge, whereas only 27.19% had good knowledge and more than one‐fifth (22.37%) exhibited poor knowledge.

Overall attitudes toward SRH were largely unfavorable. Nearly two‐thirds of adolescents (62.28%) demonstrated unfavorable attitudes toward SRH, whereas only 37.72% expressed favorable attitudes.

### 3.3 Predictors of SRH Attitudes

Table 3 presents the univariate and multivariable logistic regression analyses identifying factors associated with favorable SRH attitudes among adolescents. In the unadjusted analysis, adolescents with good and moderate knowledge of abortion were significantly more likely to have favorable SRH attitudes compared with those with poor knowledge (good knowledge: UOR = 4.39, 95% CI: 1.56–12.37; moderate knowledge: UOR = 4.99, 95% CI: 1.80–13.81). Similarly, good knowledge of STIs was associated with favorable attitudes in the univariate model (UOR = 3.64, 95% CI: 1.10–12.02).

**TABLE 3:** Univariate and multivariate logistic regression to identify predictors of SRHR attitudes (*n* = 228).

Characteristics	Unadjusted odds ratio	Confidence interval	*p* value	Adjusted odds ratio	Confidence interval	*p* value
**Knowledge about pubertal changes**						
Poor knowledge	Ref.					
Good knowledge	1.88	0.81–4.32	0.140	0.69	0.22–2.14	0.517
Moderate knowledge	1.55	0.72–3.31	0.263	0.72	0.28–1.89	0.507
**Knowledge about abortion**						
Poor knowledge	Ref.					
Good knowledge	4.39	1.56–12.37	0.005	4.41	1.09–17.83	0.037
Moderate knowledge	4.99	1.8–13.81	0.002	4.93	1.35–17.95	0.016
**Knowledge about sexually transmitted infections**						
Poor knowledge	Ref.					
Good knowledge	3.64	1.1–12.02	0.034	2.05	0.39–10.63	0.395
Moderate knowledge	2.46	0.78–7.69	0.123	1.31	0.29–5.79	0.726
**Knowledge about family planning**						
Poor knowledge	Ref.					
Good knowledge	1.41	0.66–3.03	0.373	0.78	0.28–2.16	0.626
Moderate knowledge	1.02	0.51–2.02	0.965	0.62	0.28–1.40	0.254
**Age group**						
Early adolescent	Ref.					
Late adolescent	1.39	0.80–2.42	0.247	1.45	0.76–2.78	0.262
**Grade**						
8^a^	Ref					
9	1.09	0.53–2.23	0.822			
10	0.81	0.43–1.54	0.528			
**Gender**						
Female	Ref.					
Male	0.85	0.50–1.46	0.563	0.89	0.49–1.63	0.709
**Ethnicity**						
Brahmin/Chhetri	Ref.					
Dalits	1.36	0.65–2.84	0.416	1.58	0.66–3.81	0.306
Janajatis	0.88	0.47–1.64	0.689	1.24	0.61–2.53	0.559
**Education of mother**						
Illiterate	Ref.					
Primary education	0.89	0.39–2.05	0.785	1.07	0.40–2.85	0.891
Secondary education	1.61	0.72–3.62	0.250	2.31	0.83–6.48	0.111
SLC and above	1.68	0.70–4.03	0.247	2.62	0.80–8.53	0.111
**Education of father**						
Illiterate	Ref.					
Primary education	0.86	0.27–2.69	0.788	0.71	0.17–2.90	0.635
Secondary education	0.97	0.32–2.90	0.949	0.57	0.14–2.29	0.426
SLC and above	1.25	0.40–3.86	0.698	0.72	0.17–3.16	0.667

^a^ Removed from the multivariable logistic regression because of high correlation with age.

After adjusting for potential confounders including age, gender, ethnicity, and parental education, knowledge of abortion remained the only SRH knowledge domain independently associated with favorable SRH attitudes. Adolescents with good abortion knowledge had more than four times higher odds of favorable attitudes compared with those with poor knowledge (AOR = 4.41, 95% CI: 1.09–17.83), whereas those with moderate knowledge also showed significantly higher odds (AOR = 4.93, 95% CI: 1.35–17.95). Associations observed for knowledge of pubertal changes, STIs, and family planning were no longer statistically significant in the adjusted model.

None of the sociodemographic variables, including age group, gender, ethnicity, grade level, or parental education, were significantly associated with SRH attitudes in either unadjusted or adjusted analyses. Grade level was excluded from the multivariable model due to collinearity with age.

## 4 Discussion

This study examined SRH knowledge and attitudes among adolescents attending government schools in Tansen Municipality, Palpa, Nepal. The findings demonstrate persistent gaps in SRH knowledge across all assessed domains, which include pubertal changes, abortion, STIs, and family planning. Similarly, a predominance of unfavorable SRH attitudes was observed among adolescents. These findings have important implications for adolescent health programming and school‐based sexual health education in Nepal.

Although most adolescents demonstrated moderate levels of knowledge, relatively few achieved good knowledge across SRH domains, whereas poor knowledge remained common, particularly regarding family planning and abortion. This pattern suggests that current school‐based SRH education introduces basic concepts but may not sufficiently equip adolescents with comprehensive and practical understanding. Similar gaps have been reported in school‐based studies from Surkhet and Chitwan districts of Nepal, indicating that limited depth and inconsistent implementation of SRH curricula remain systemic challenges [14, 15].

Despite the formal inclusion of comprehensive sexuality education (CSE) in Nepal's national curriculum, sociocultural and institutional barriers continue to constrain effective delivery. Sexuality‐related topics are often considered sensitive or inappropriate for open discussion, and teachers may not deliver the content appropriately with the potential feeling of embarrassment when addressing SRH content [16, 17]. In addition, structural barriers such as insufficient teaching time, lack of audiovisual materials, and inadequate teacher training further could limit the opportunities for meaningful student engagement [14, 16]. Evidence from South Asia and other LMICs suggests that SRH knowledge alone may be insufficient to translate into positive SRH behaviors without supportive educational environments that encourage dialogue and critical thinking [17, 26, 27].

Teachers were commonly reported as important sources of SRH information in Nepal and other LMIC settings, highlighting schools as a critical entry point for adolescent SRH interventions [28, 29]. However, teacher–student interactions around SRH were often characterized by discomfort and superficial content delivery, with limited focus on rights‐based or practical issues [16, 17]. Strengthening teacher capacity through targeted training and promoting participatory teaching methods, such as group discussions and role‐play, may enhance the delivery of school‐based SRH education [30]. Additionally, digital and media‐based interventions may complement classroom teaching by providing adolescents with confidential access to accurate information [31, 32].

A key finding of this study is the strong and independent association between abortion‐related knowledge and favorable SRH attitudes. After adjusting for sociodemographic factors and other SRH knowledge domains, adolescents with moderate or good knowledge of abortion were significantly more likely to report favorable attitudes. This aligns with global evidence indicating that awareness of legal and safe abortion is associated with more progressive SRHR attitudes among adolescents [2]. However, the wide CI on the odds ratio (95% CI: 1.09–17.83) suggests wide margin of error and statistical uncertainty of the estimate. This wide margin depicts that the exact strength of the relationship could vary. However, the direction of the relationship is stable and showing that having good abortion‐related knowledge correlates with positive SRH attitude. The wide CIs could be due to the limited sample size in each categories of abortion knowledge. The absence of significant associations for other SRH knowledge domains may suggest that general factual SRH knowledge alone is not always linked with favorable attitudes, unless the information also covers practical and commonly stigmatized issues, such as abortion, contraception, and adolescent sexual health decision‐making [33, 34].

The high prevalence of unfavorable SRH attitudes observed in this study is concerning, given that attitudes are commonly linked with health‐seeking behavior and service utilization [35, 36]. Comparable findings have been reported in studies from Iran and Ethiopia, indicating that negative attitudes toward SRH remain common in settings where sexuality education is constrained by cultural norms [37].

These findings point to several clear steps for improving adolescent SRH programs and policies in Nepal. First, CSE should be strengthened by ensuring that important topics, such as abortion, contraception, and puberty, are taught in a scientifically accurate manner that is appropriate to students’ age and maturity level and emphasizes safe practices, informed decision‐making, and respect for adolescents’ health needs. Moreover, school‐based SRH education should place greater emphasis on practical and actionable information, such as pregnancy prevention, safe abortion awareness, and STI prevention, as well as guidance on where adolescents can access youth‐friendly SRH services, rather than focusing primarily on biological descriptions of the reproductive system. Second, teacher training could be provided, and that should focus on increasing educators’ confidence and skills to deliver SRH lessons through interactive approaches, such as group discussions, case studies, and question‐and‐answer sessions. Likewise, those sessions will also be helping teachers address stigma and cultural discomfort. Third, schools should build stronger connections with adolescent‐friendly health services through referral systems and regular outreach sessions led by trained health workers, improving access to confidential counseling and care. Finally, community engagement should include parents, local leaders, and youth groups to encourage open discussion of SRH and reduce taboos that prevent adolescents from receiving accurate information. Together, these strategies could reduce ongoing knowledge gaps and support healthier SRH attitudes and behaviors among adolescents.

This study has several limitations. The cross‐sectional approach of this study means that no causal relationships were established regarding SRH knowledge and SRH attitudes. Another limitation of the study relates to the use of self‐reporting. As SRH‐related information and questions are very personal in nature, this could have caused social desirability bias. Another limitation concerns the internal consistency of the measurement tools, as several SRH knowledge domains and the attitude scale showed low reliability coefficients. In particular, the SRH attitude scale demonstrated poor internal consistency (Cronbach's *α* = 0.48), indicating potential measurement error and possible misclassification of favorable attitudes. Such misclassification may have weakened true associations and reduced the precision of the logistic regression estimates, leading to an underestimation of the relationship between SRH knowledge and attitudes. Therefore, results related to predictors of SRH attitudes should be interpreted with caution. Further research is recommended to refine and validate these scales using psychometric methods to develop more accurate and reliable measures for Nepalese adolescents. Additionally, although percentage scoring was applied to allow comparison across SRH knowledge domains, differences in item counts may have influenced score distribution. Finally, the purposive selection of only three government schools may have introduced selection bias and limited the generalizability of the findings. The selected schools may differ from other schools in terms of educational environment, SRH teaching practices, and student characteristics. Therefore, the findings may not fully represent all adolescents in Nepal.

## 5 Conclusion

This study reveals major gaps in adolescents’ understanding of SRH, especially around family planning, puberty‐related changes, and abortion. Most participants also showed negative attitudes toward SRH. Notably, better knowledge about abortion was strongly linked to more positive SRH attitudes.

Overall, the findings point to the need for stronger school‐based CSE and focused adolescent SRH programs. These efforts should prioritize accurate information on abortion and other key SRH topics, supported through improved teacher training and better connections to adolescent‐friendly health services.

## Author Contributions

S.B. and S.P. contributed to the conceptualization, methodology, data collection, analysis, writing, review, and editing. K.P.S. and N.A. were responsible for the methodology, data analysis, results interpretation, writing, review, and editing. R.B.R., R.P., B.A., and B.T. contributed to the methodology, writing, review, data editing and editing.

## Funding

The authors have nothing to report.

## Consent

The authors have nothing to report.

## Conflicts of Interest

The authors declare no conflicts of interest.

## Supporting information




**Supporting File**: puh270305‐sup‐0001‐SuppMat.pdf

## Data Availability

The datasets and/or analyzed during the current study are available from the corresponding author upon reasonable request. These data are not publicly accessible in order to protect participant confidentiality.
