# Supplementary material for: Knowledge and Attitudes Toward Sexual and Reproductive Health Among Adolescents Studying in Government Schools in Tansen Municipality, Palpa, Nepal: A School‐Based Cross‐Sectional Study
Source: Public Health Chall. 2026 Jun 23;5(2):e70305. doi: 10.1002/puh2.70305 (PMC13288163; doi:10.1002/puh2.70305)
Supplement: Supplementary file 1 — Supporting File: puh270305‐sup‐0001‐SuppMat.pdf [file PUH2-5-e70305-s001.pdf]

Knowledge and Attitudes Toward Sexual and Reproductive Health Among Adolescents Studying in Government Schools in Tansen Municipality, Palpa, Nepal: A School Based Cross-Sectional Study

The questionnaire has three parts.

Direction: Put tick marks (✓) for appropriate answer and fill the answer in the blank space.

Code no: -   Date:-

Part I- Questions related to socio demographic information

1. Age ..... (in completed years)
2. Sex
   a) Male   c) others
   b) Female

3. Class......

4. Ethnicity
   a. Dalit
   b. Disadvantaged Janajatis (Magar, Rai, Limbu, Sherpa)
   c. Disadvantaged non dalit Terai caste group (Yadav, Teli)
   d. Religious minorities (Muslim, Chureto)
   e. Relatively advantaged Janajatis (Newar and Thakali)
   f. Upper caste group (Bramhin, Chhetri, Thakuri, Rajput, Madwaadi)

5. Parent's education

Father	Mother
a) Illiterate	a) Illiterate
b) Primary	b) Primary
c) Secondary	c) Secondary
d) SLC and above	d) SLC and above

6. From where did you get information about Adolescent Sexual and reproductive health? (Multiple response)
   a) Radio/ television   d) Sibling
   b) School teachers   e) Peers
   c) Parents education

Part II – Knowledge on Sexual and Reproductive Health

Section 'A'- Pubertal Changes

1) The physical transformation from child to adult is called
   a) Puberty   c) Young adult
   b) Middle adult   d) Elderly
   c)

2) What are the changes seen in adolescent? (Multiple response)

a) Physical
b) Cognitive
c) Sexual
d) Emotional
e) Social

3) What are the causes for pubertal changes?

a) Hormonal
b) Non – hormonal
c) Disease
d) Age

4) What are the pubertal changes in boys? (Multiple response)

a) Development of testes and scrotum.
b) Attraction to opposite sex.
c) Night falling.
d) Hair growth in under arms, legs and genital area.
e) Voice may crack.

5) What are the pubertal changes in girls? (Multiple response)

a) Breast develop
b) Hair growth in under arms and genital area.
c) Menstruation cycle
d) White discharge from vagina
e) Height increases, longer extremities

Section – 'B' Abortion

6) What do you mean by abortion?

a) Termination the product of conception before the completion of 22 weeks.
b) Termination of the product of conception after 28 week.
c) Termination of the product of conception after 30 weeks.
d) Termination of the product of conception before completion of 37 weeks

7) Has Nepal government legalized the safe abortion?

a) Yes
c) No

8) If yes, up to how many weeks of pregnancy can be terminated voluntarily?

a) 10 weeks
b) 12 weeks
c) 18 weeks
d) 22 weeks

9) What do you mean by unsafe abortion?

a) Performed abortion by untrained person
b) Performed abortion by trained person
c) Termination of pregnancy after 22 weeks
d) Termination of pregnancy before 28 weeks.

10) Who goes towards unsafe abortion? (Multiple response)

a) Unmarried girls
b) Married women
c) People who had no knowledge about safe abortion
d) Unwanted pregnancy
e) Rape Case

11) Why do young girls mostly undergo unsafe abortion? (Multiple response)
   a) Lack of money
   b) Hesitation to go medical Centre
   c) Fear of exposure to others
   d) Lack of knowledge
   e) Unwanted pregnancy

12) What are the complications of unsafe abortion? (Multiple response)
   a) Heavy bleeding
   b) Incomplete abortion
   c) damage to reproductive organs
   d) Infection of uterus
   e) Infertility

13) How can be prevented the unsafe abortion? (Multiple response)
   a) Awareness program
   b) Proper counselling
   c) Strict law
   d) Proper monitoring
   e) Incorporate into the curriculum

Section – 'C' Sexually Transmitted Infections

14) What is sexual transmitted infection (STIs)?
   a) It is an infection of urinary tract.
   b) It is an infection of genital tract.
   c) It is an infection of oral canal.
   d) It is an infection of skin.

15) What are the causes of STIs? (Multiple response)
   a) Bacteria
   b) Virus
   c) fungus
   d) Protozoa

16) What are the different types of STIs? (Multiple response)
   a) Syphilis
   b) Gonorrhea
   c) HIV/AIDS
   d) Trichomoniasis
   e) Chlamydia

17) What are the modes of transmission of STIs? (Multiple response)
   a) Unsafe intercourse
   b) Unsafe blood transfusion
   c) From infected mother to child during child birth
   d) Sharing infected needles
   e) Multiple sex partners

18) What are the signs and symptoms of STIs? (Multiple response)
   a) Whitish discharge from penis and vagina
   b) Rashes around genital area
   c) Genital itching
   d) Painful urination
   e) Pain during intercourse

19) What are the preventative measures of STIs? (Multiple response)
   a) Discouraging unsafe sexual behavior.
   b) Use of condom during sex.
   c) Maintain good genital hygiene.
   d) No exchange of injected needles.
   e) Check blood before transfusion.

20) What are the common complications of STIs? (Multiple response)
   a) Infertility
   b) Still birth
   c) Cervical cancer
   d) Miscarriage
   e) Congenital Anomalies

Section – 'D' Family Planning

21) What do you mean by family planning?
   a) The practice of controlling number of children birth.
   b) The practice of infertility.
   c) The practice of having more children.
   d) The practice of abstinence in sex.

22) Why family planning methods are used? (Multiple response)
   a) To have children as needed.
   b) To space childbirth.
   c) To improve the health of mother and children.
   d) To make happy family.
   e) To prevent from unwanted pregnancy.

23) What methods of family planning have you heard about? (Multiple response)
   a) Permanent
   - Minilap
   - Laparoscopy
   - Vasectomy

   b) Temporary
   - Oral contraceptives pills
   - Depo- Provera
   - Implant
   - Copper –T
   - Natural
   - Other specify……

24) What do you mean by emergency contraception?
   a) Contraceptive which is taken within 72 hours of unprotected sex.
   b) Contraceptive which is taken within 7 days of unprotected sex.
   c) Contraceptives which is taken within 5 days of menstruation.
   d) Contraceptives which is taken regular after having sex.

Part III – Attitude of Sexual and Reproductive Health

Direction: express your opinion by agreeing on statement that you feel in correct and by disagreeing to the statement that you feel is incorrect.

S.N	Statements	Strongly disagree	Disagre e	Neutral	Agree	Strongly agree
1	Adolescent, single or married should know how to use contraceptives.					
2	Educational booklets about pregnancy and STIs/ AIDS prevention methods should be available in communities					
3	The best method for prevention of STIs/ AIDS is abstinence until marriage.					
4	Contraceptives use for a long period cause infertility.					
5	Providing emergency contraceptives pills would discourage consistent use of other contraceptives.					
6	Contraceptives including condoms should available easily to adolescent.					
7	Education about safe abortion, pregnancy and STIs /HIV/AIDS prevention methods lead to high risk sexual behaviors.					
8	Friend and internet alone not provide complete information so parents also provide information regarding pubertal changes.					
9	Menstruation is a shameful and embarrassing situation for girls.					
10	Adolescent do not need Sexual and Reproductive health information because they have no any premarital intercourse.					
